# Supplementary material for: Identification of an alternative triglyceride biosynthesis pathway
Source: Nature. 2023 Aug 30;621(7977):171–8. doi: 10.1038/s41586-023-06497-4 (PMC10482677; doi:10.1038/s41586-023-06497-4)

nature portfolio

https://doi.org/10.1038/s41586-023-06497-4

Supplementary information

Identification of an alternative triglyceride biosynthesis pathway

In the format provided by the authors and unedited

Supplementary Information Figure 1 - uncropped/unprocessed Western blots

Fig. 1c

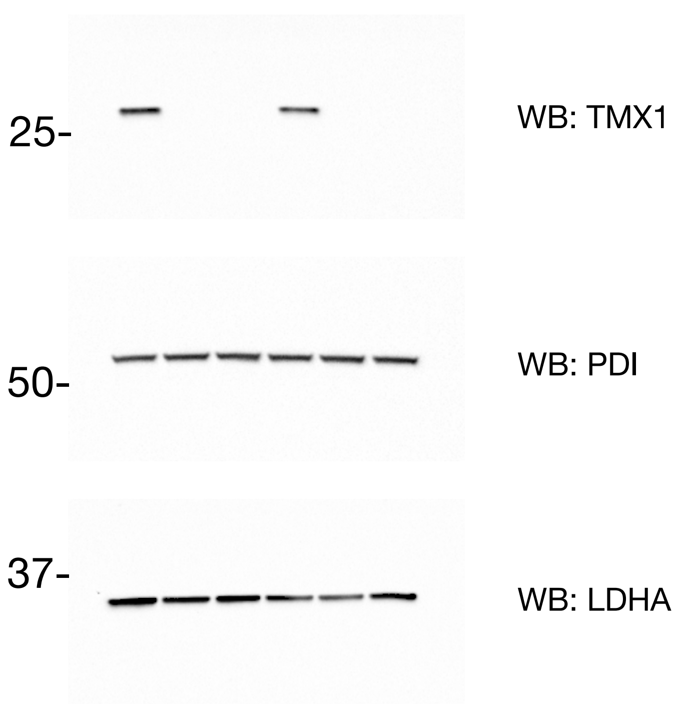

Fig. 2e

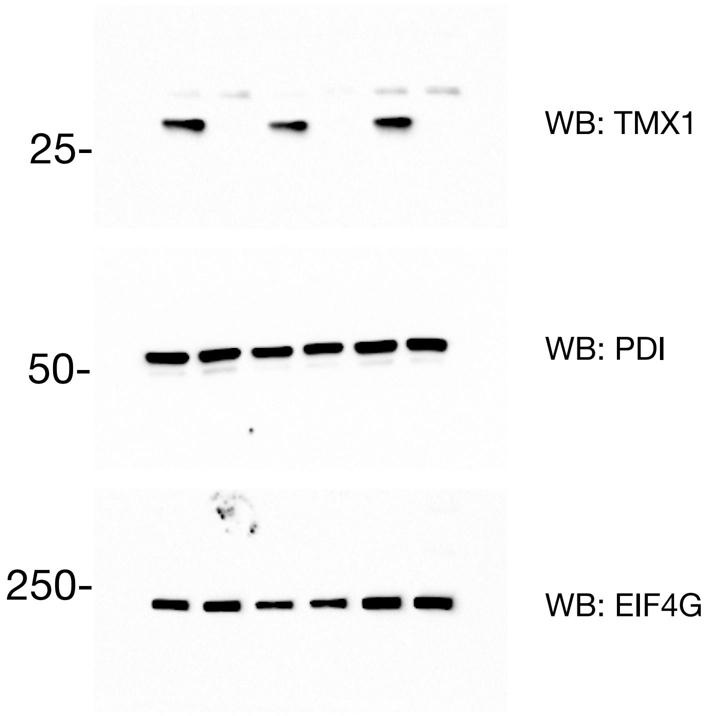

Fig. 3a

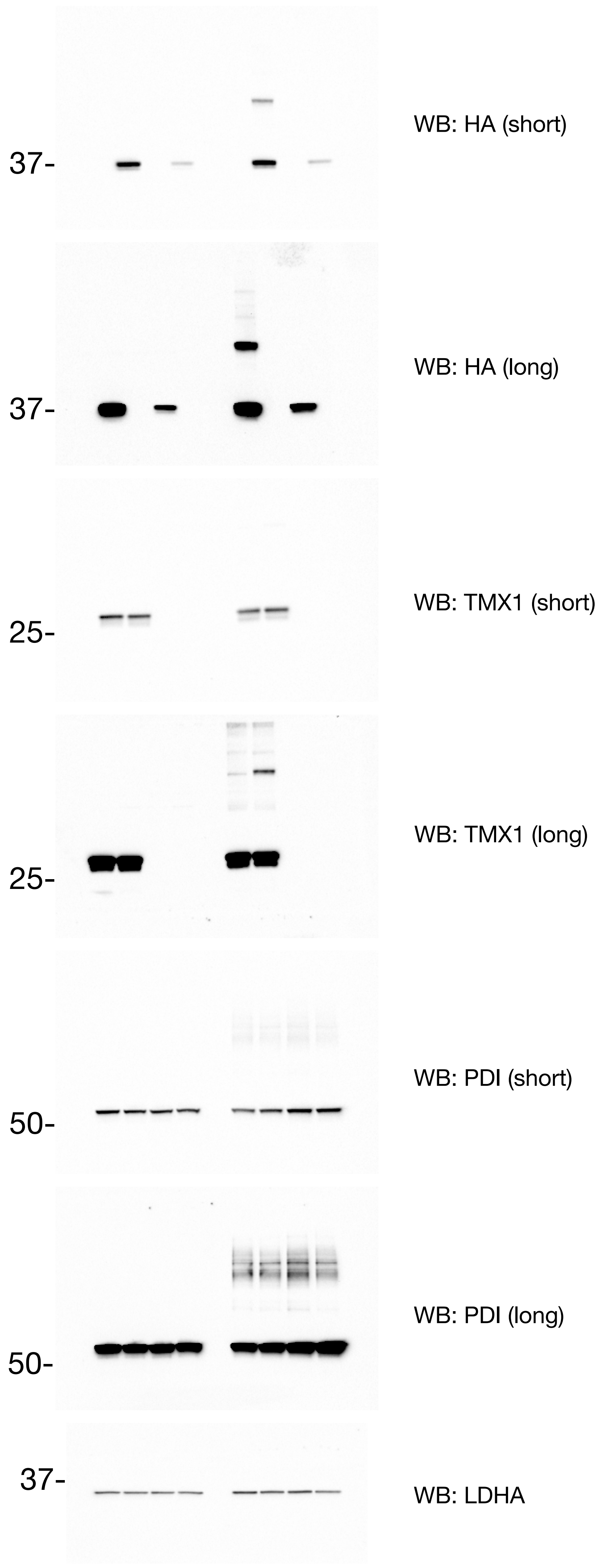

Fig. 3b

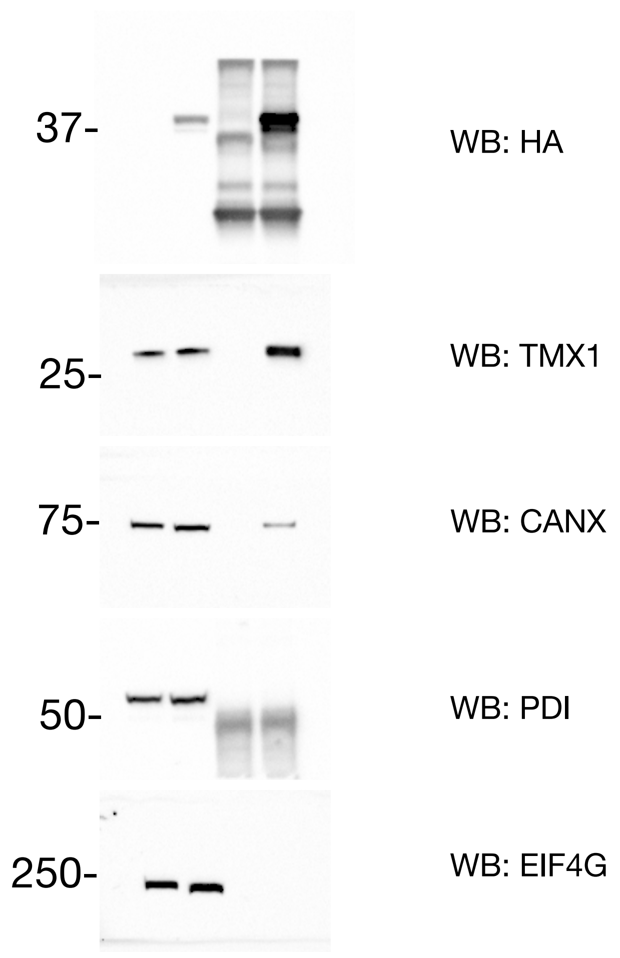

Fig. 3d

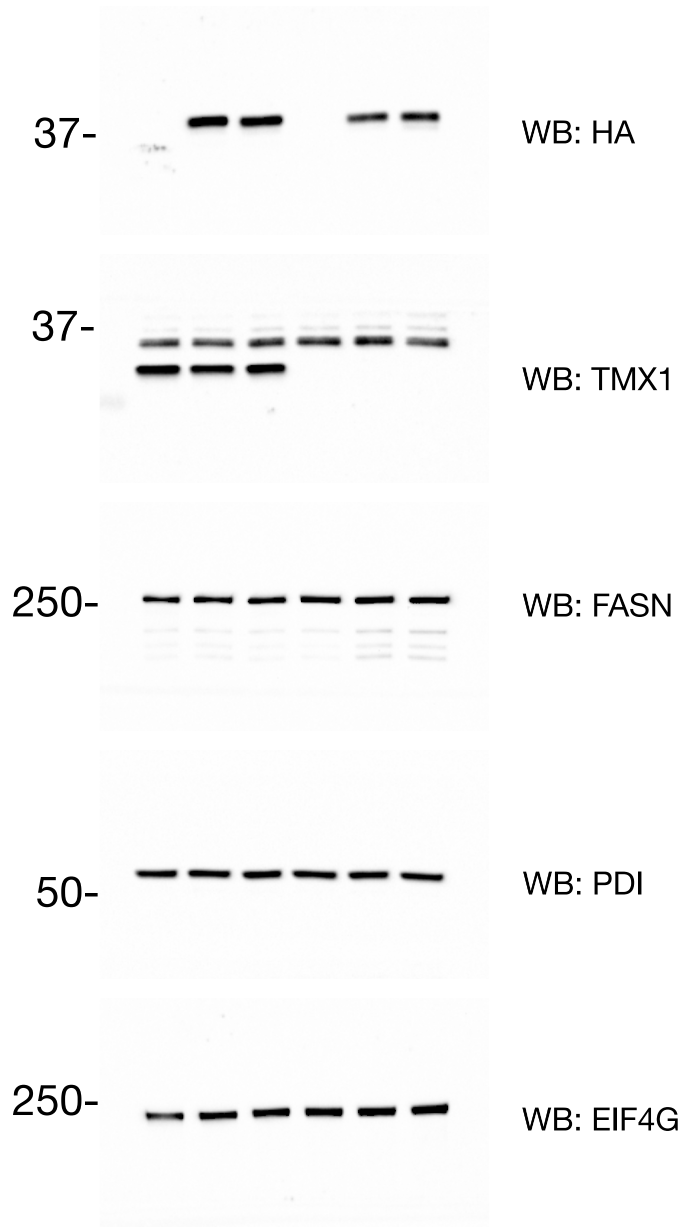

Fig. 4e

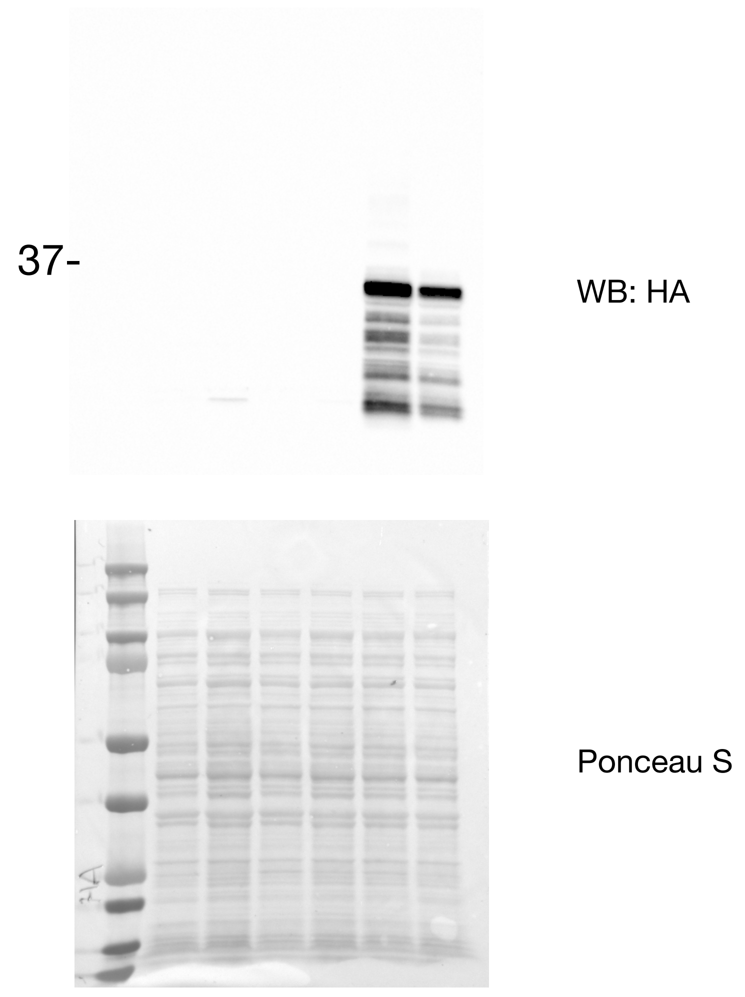

Fig. 5a

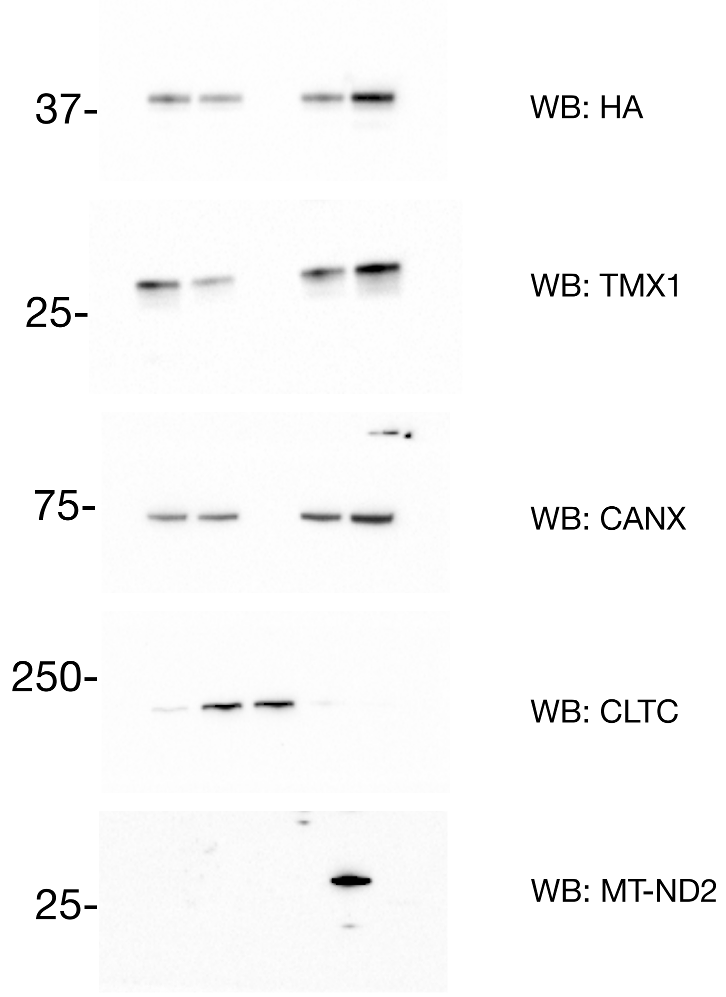

Supplementary Information Figure 1 (continued)

Extended Data Fig. 2b - A549

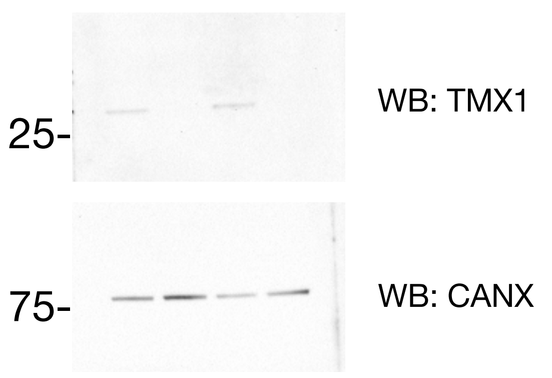

Extended Data Fig. 2b - U2OS

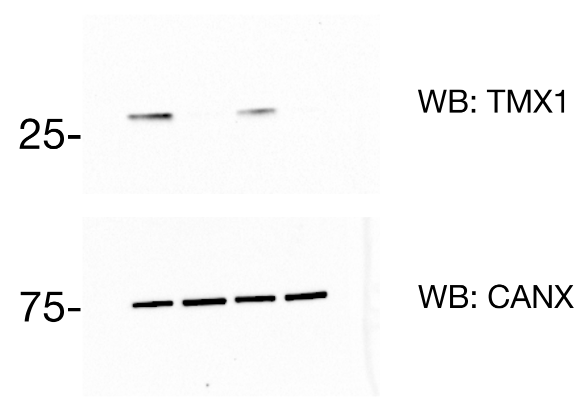

Extended Data Fig. 2e

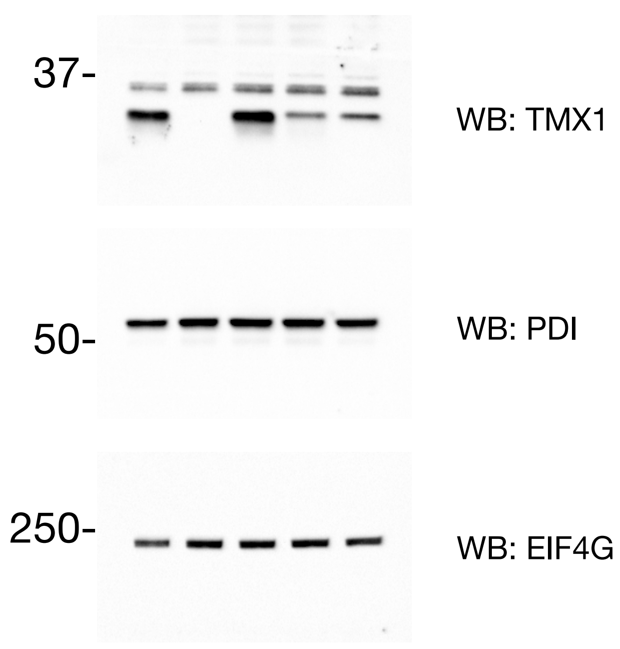

Extended Data Fig. 2i

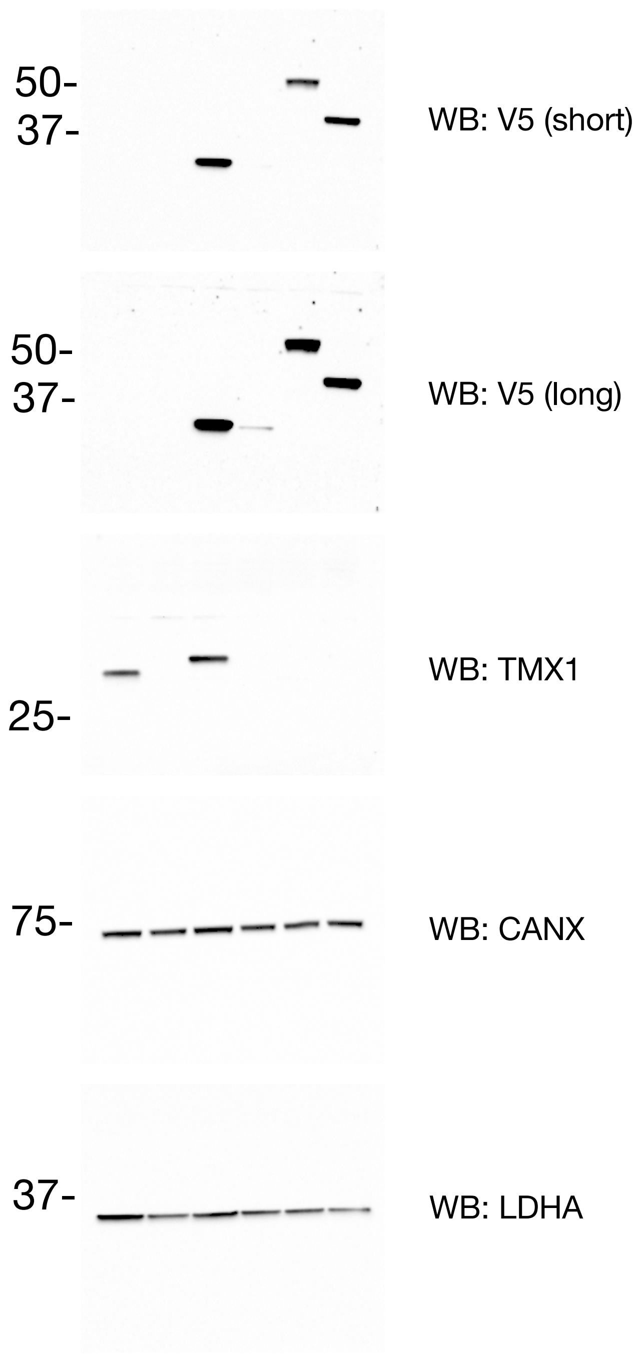

Extended Data Fig. 4b

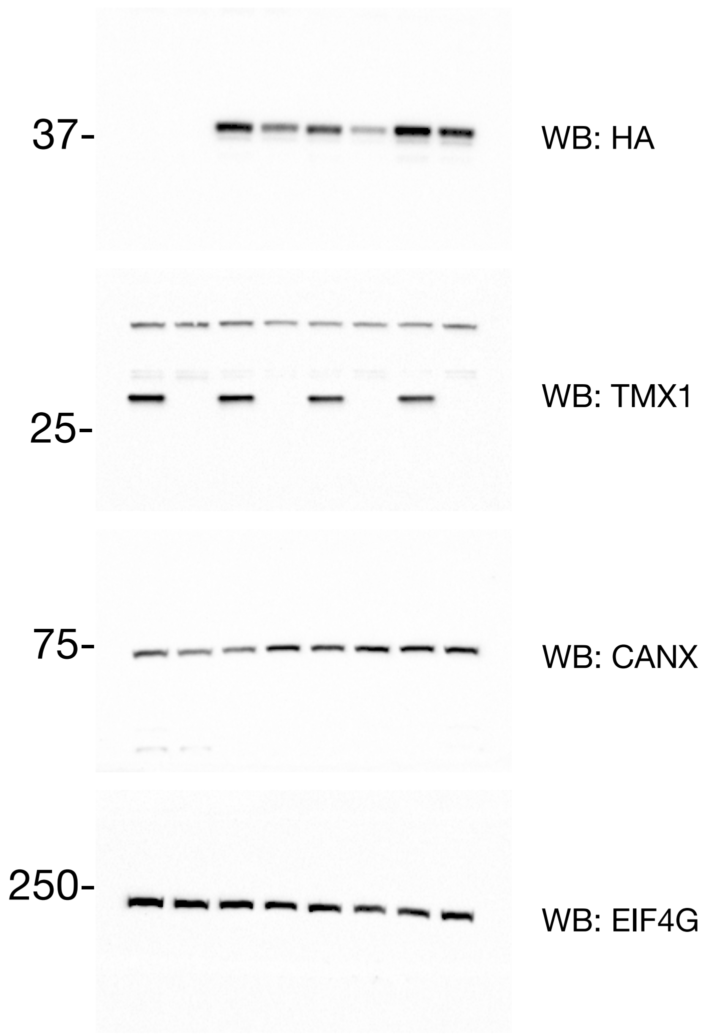

Extended Data Fig. 4c

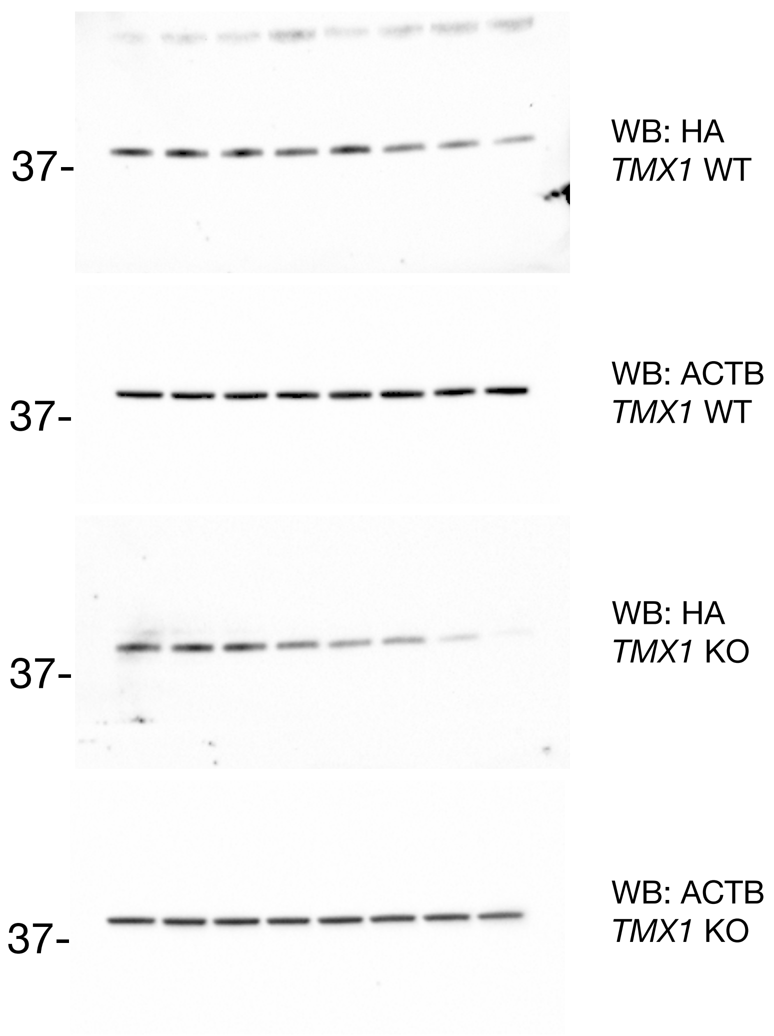

Extended Data Fig. 4d

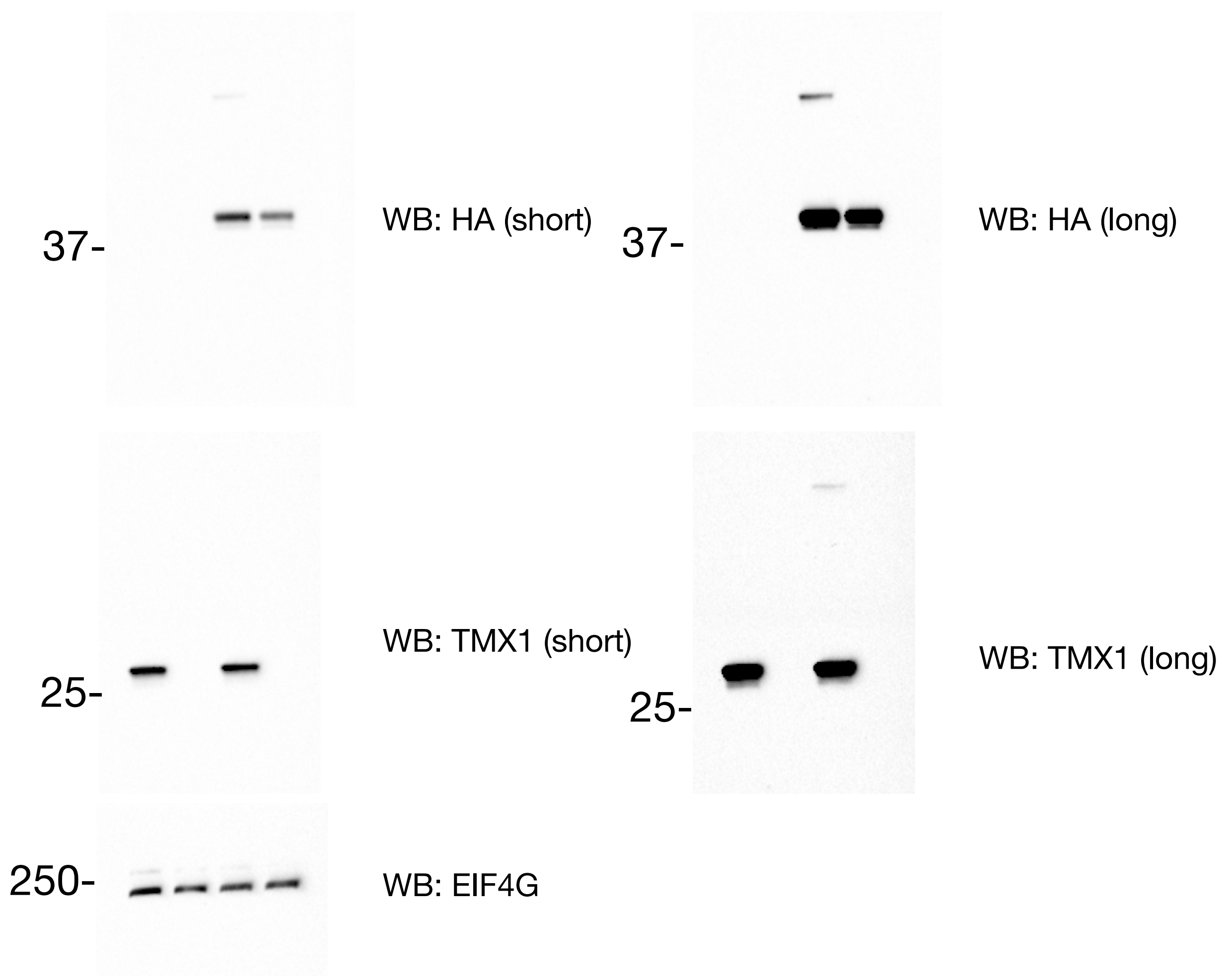

Extended Data Fig. 4f

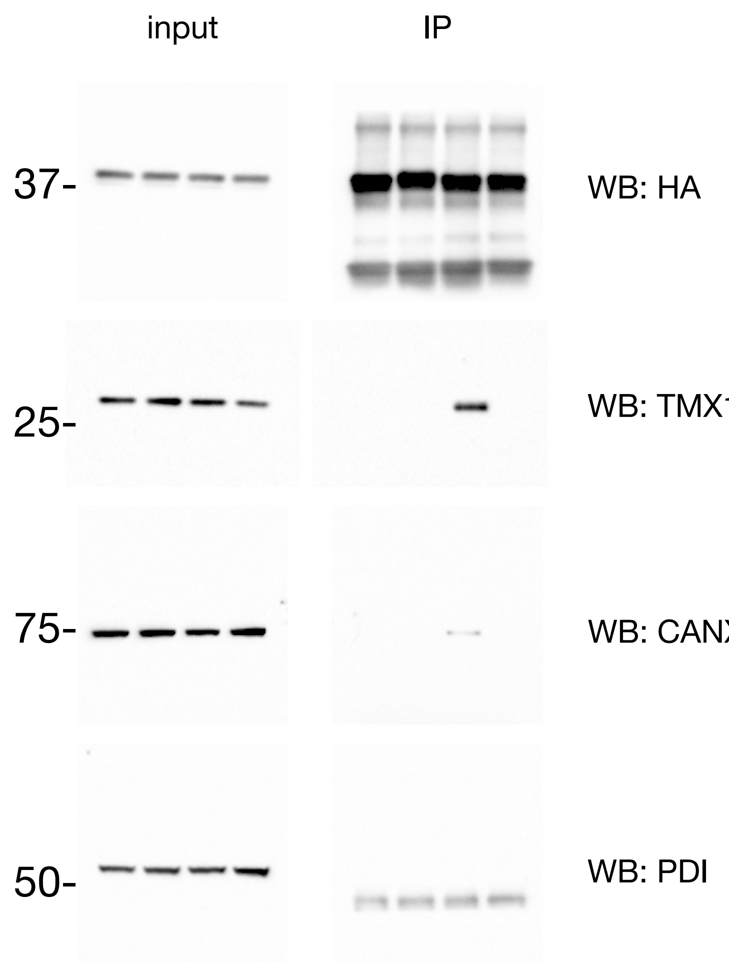

Extended Data Fig. 4h

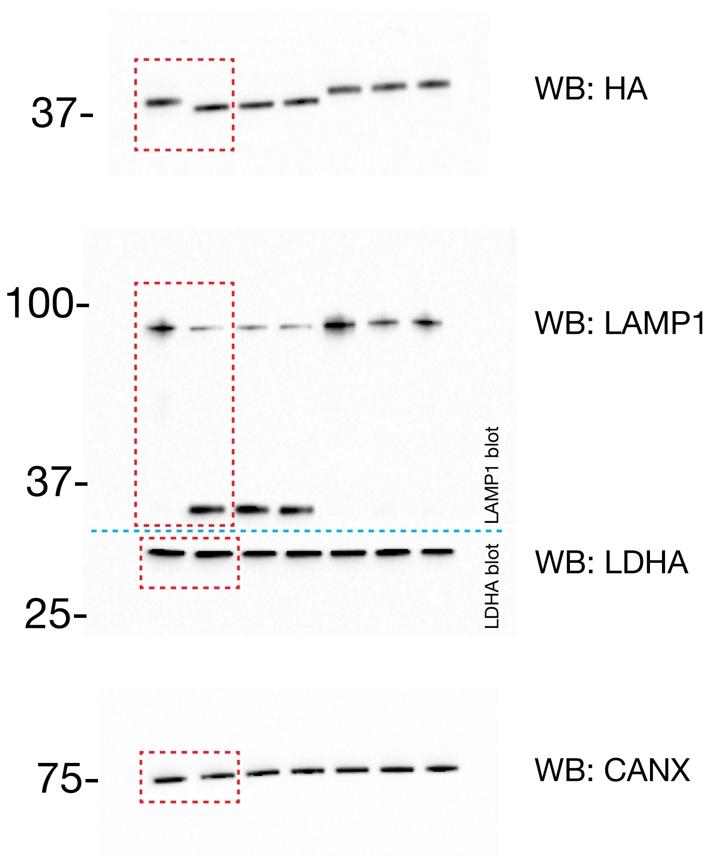

Extended Data Fig. 4i

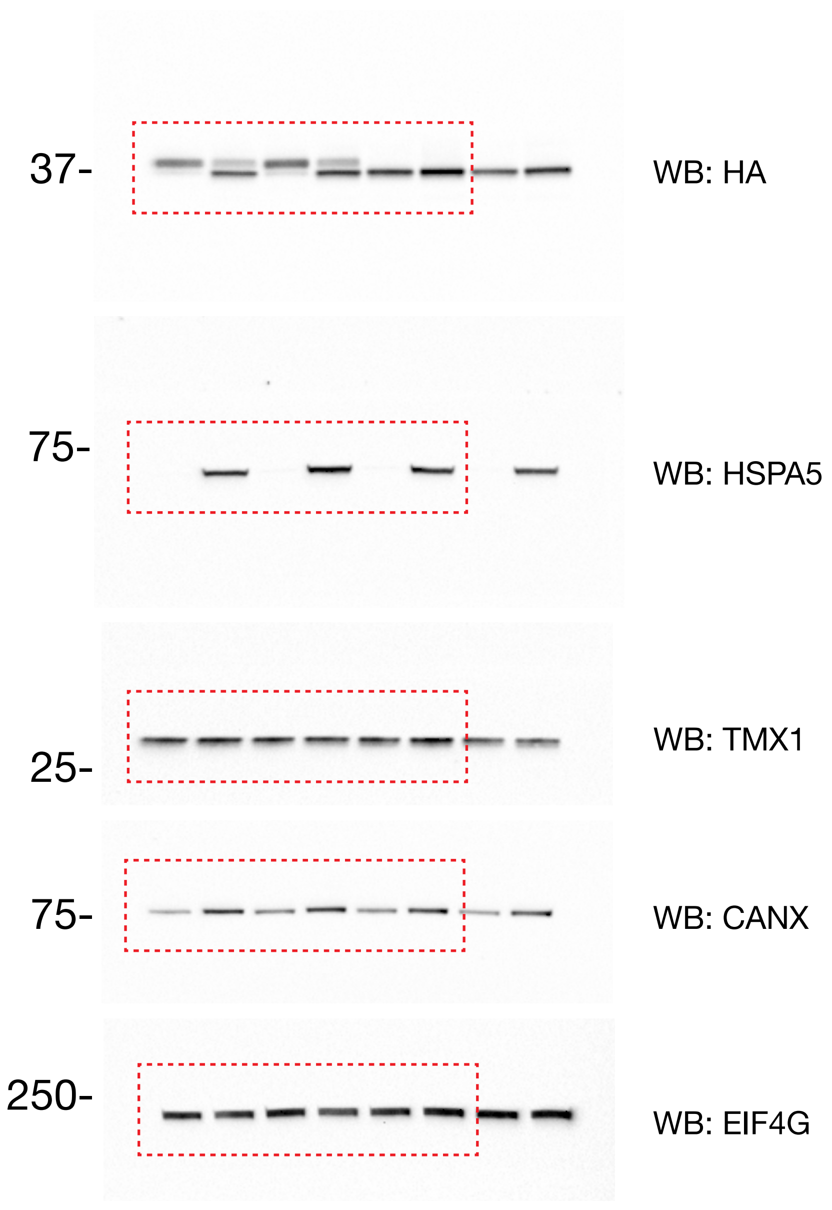

Supplementary Information Figure 1 (continued)

Extended Data Fig. 4j

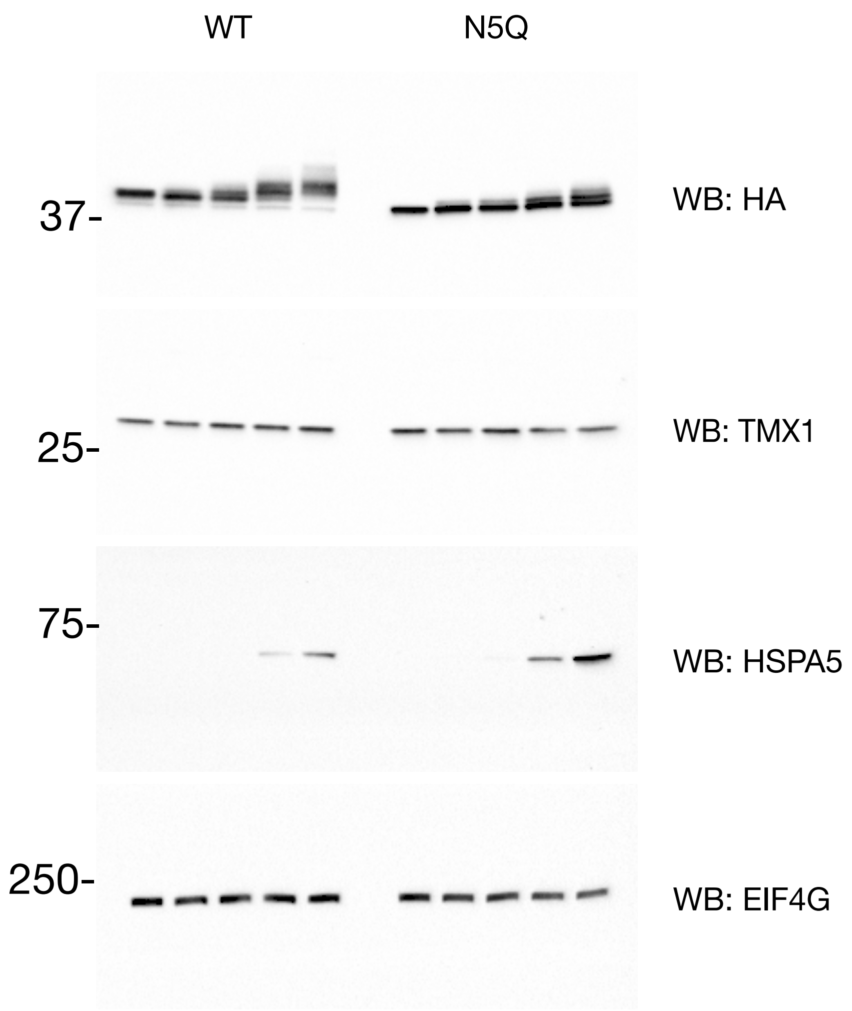

Extended Data Fig. 4k

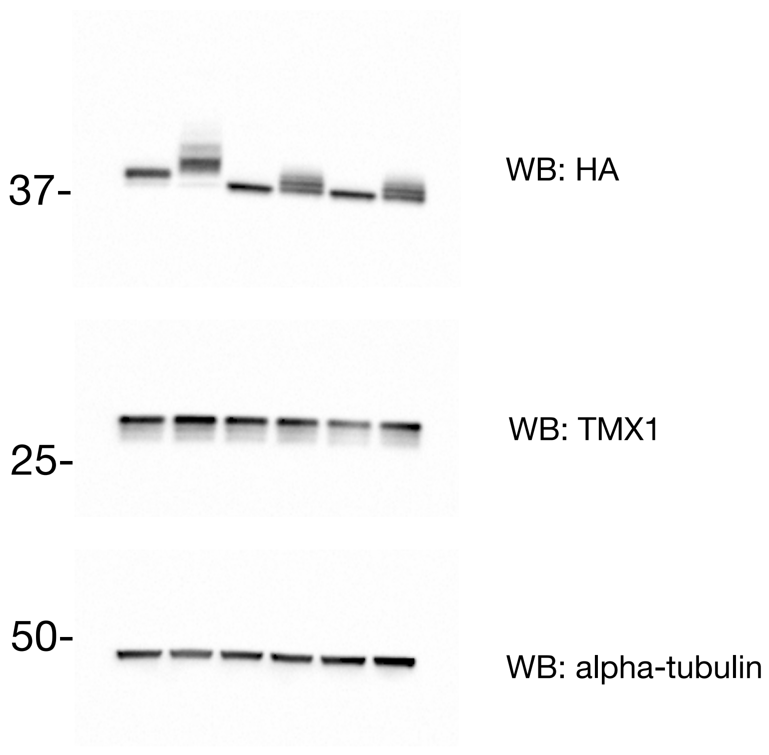

Extended Data Fig. 4o

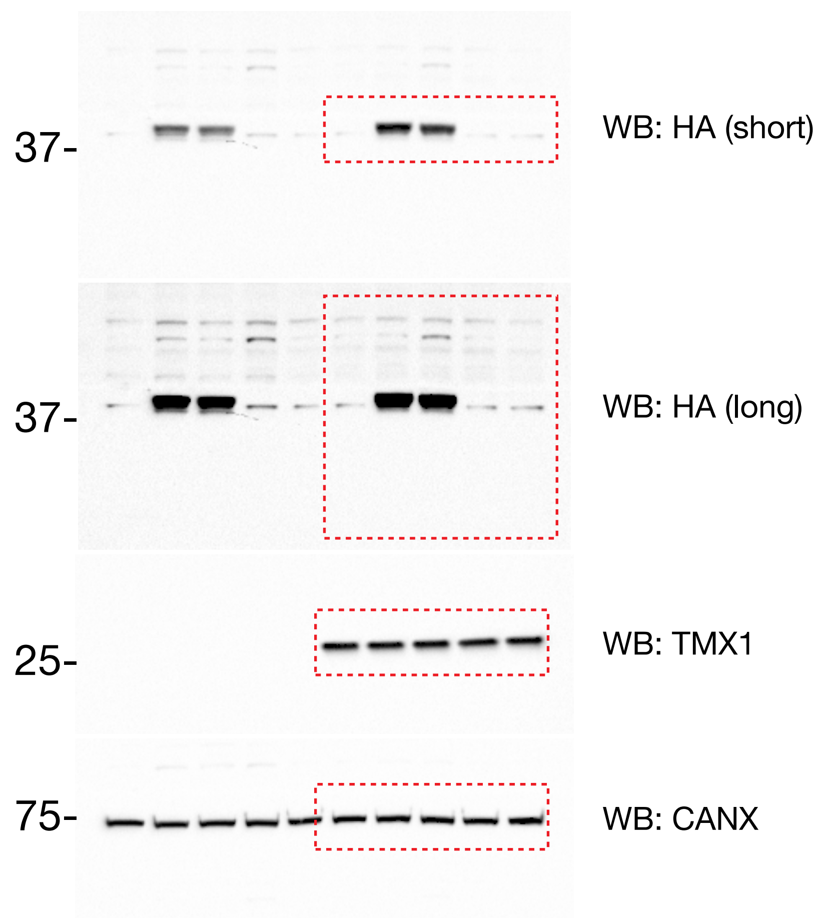

Extended Data Fig. 5b

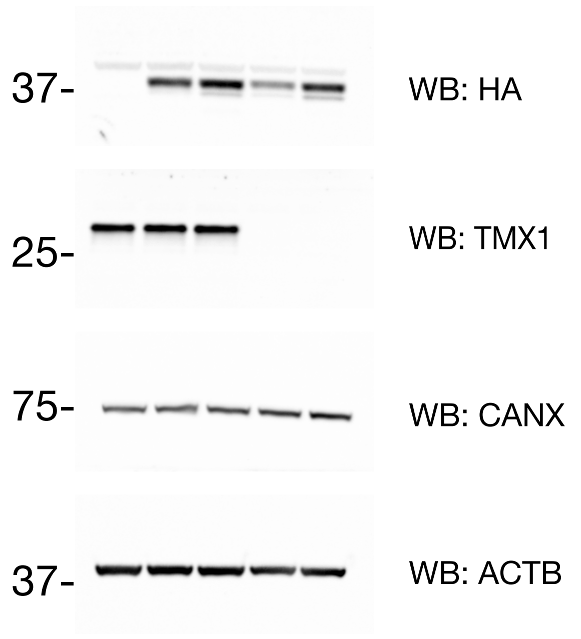

Extended Data Fig. 8a: RPE1

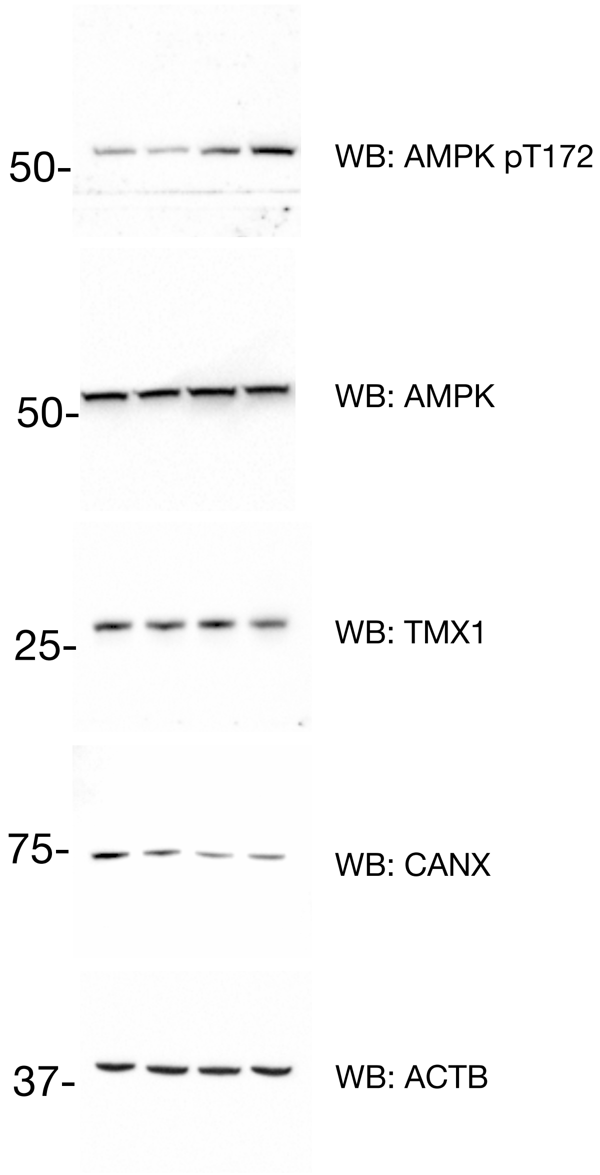

Extended Data Fig. 8a: HT29

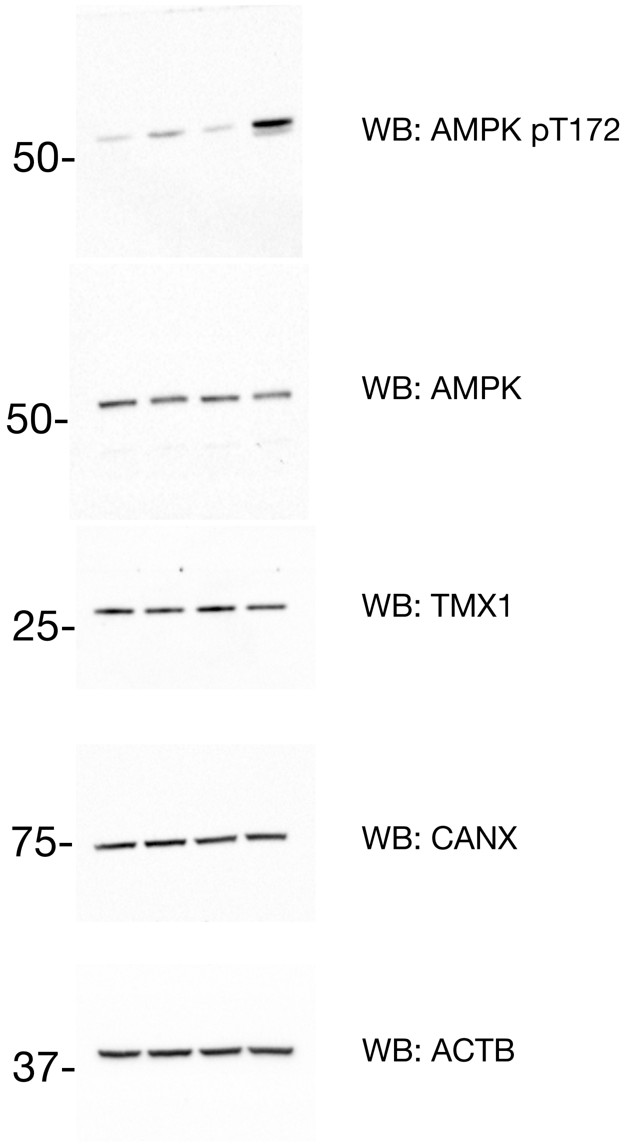

Extended Data Fig. 8a: U251

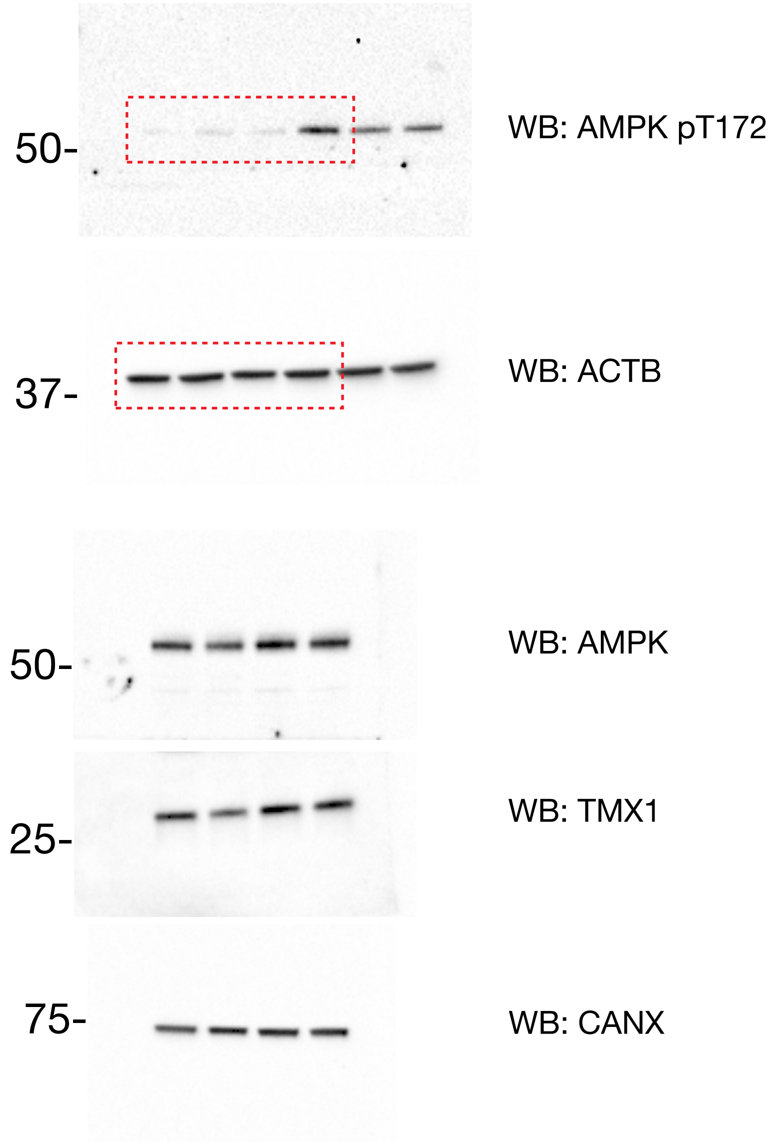

Extended Data Fig. 8b

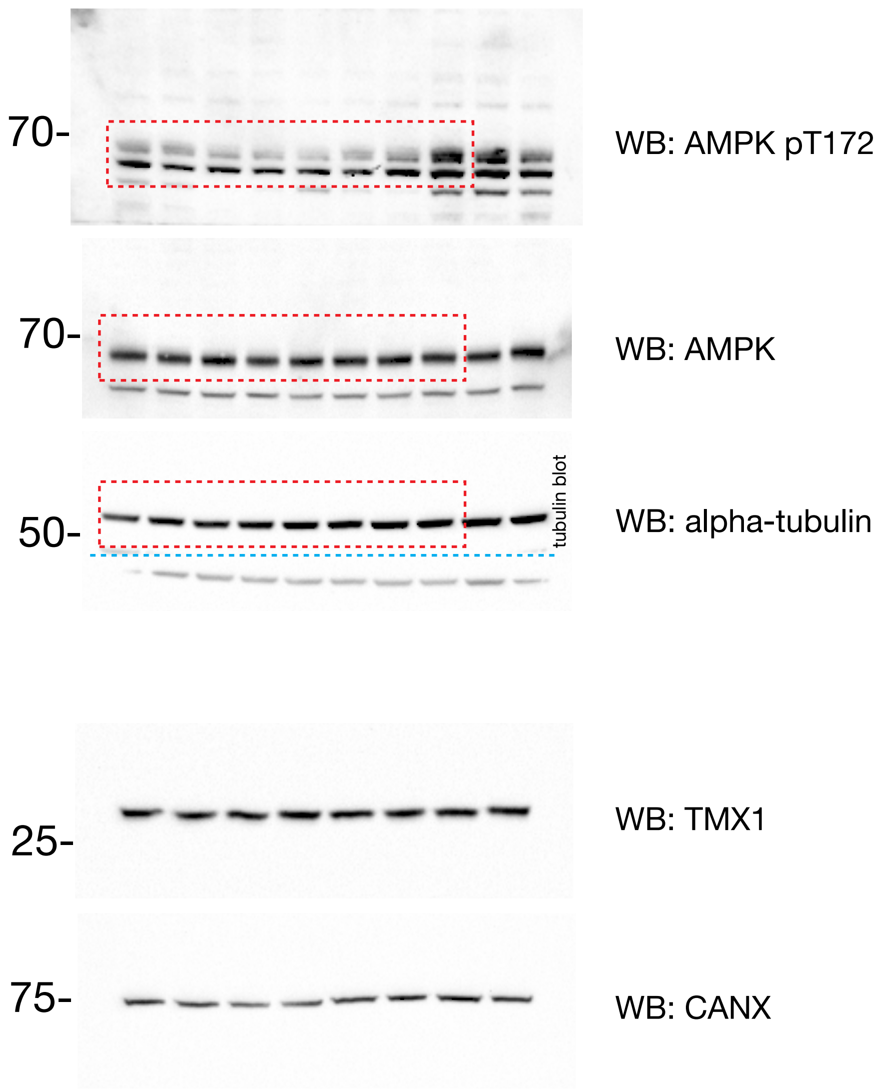

Extended Data Fig. 8d

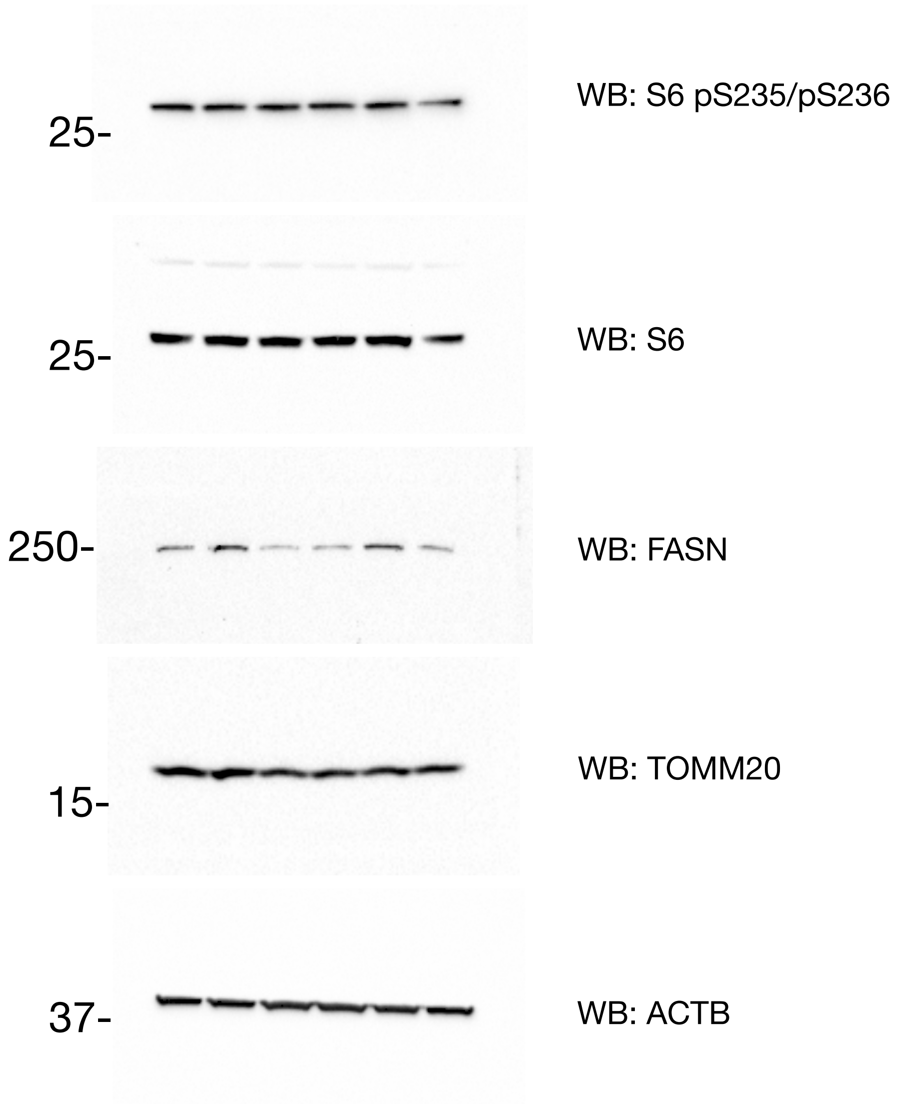

Supplementary Information Figure 2 - gating strategy for FACS and analysis

gating strategy: fixed HAP1 cells

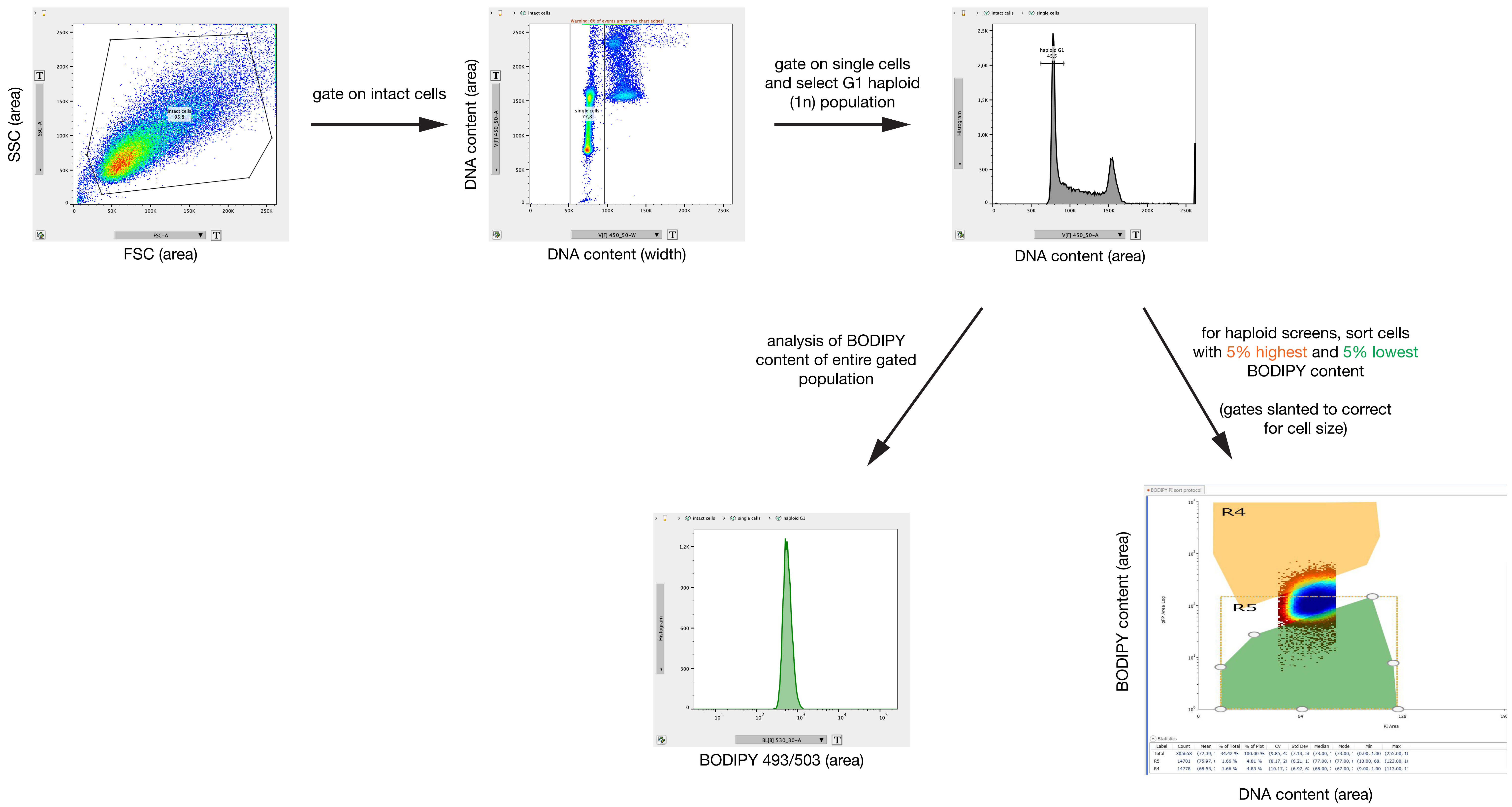

gating strategy: live RPE1 cells

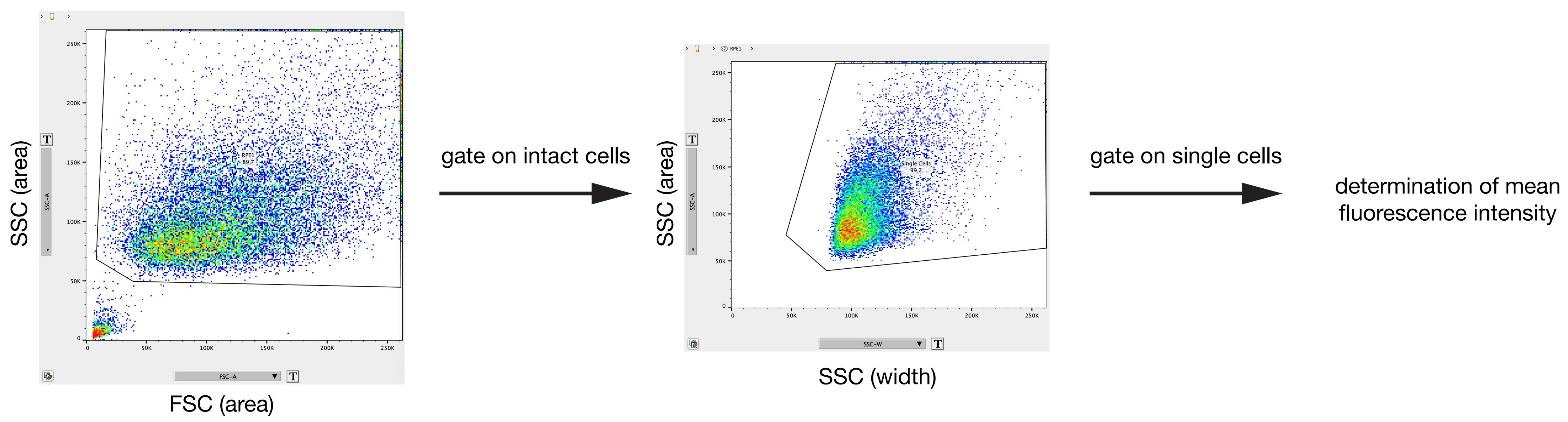

Supplement: Supplementary file 1 — Supplementary Fig. 1: uncropped and unprocessed western blots; Supplementary Fig. 2: flow cytometry gating strategy. [file 41586_2023_6497_MOESM1_ESM.pdf]
