# Supplementary material for: Identification of an alternative triglyceride biosynthesis pathway
Source: Nature. 2023 Aug 30;621(7977):171–8. doi: 10.1038/s41586-023-06497-4 (PMC10482677; doi:10.1038/s41586-023-06497-4)
Supplement: Supplementary file 2 — Reporting Summary [file 41586_2023_6497_MOESM2_ESM.pdf]

nature portfolio

Corresponding author(s): Gian-Luca McLelland, Thijn Brummelkamp

Last updated by author(s): Jul 3, 2023

Reporting Summary

Nature Portfolio wishes to improve the reproducibility of the work that we publish. This form provides structure for consistency and transparency in reporting. For further information on Nature Portfolio policies, see our Editorial Policies and the Editorial Policy Checklist.

Statistics

For all statistical analyses, confirm that the following items are present in the figure legend, table legend, main text, or Methods section.

n/a	Confirmed	
☐	☒	The exact sample size (n) for each experimental group/condition, given as a discrete number and unit of measurement
☐	☒	A statement on whether measurements were taken from distinct samples or whether the same sample was measured repeatedly
☐	☒	The statistical test(s) used AND whether they are one- or two-sided<br>*Only common tests should be described solely by name; describe more complex techniques in the Methods section.*
☐	☒	A description of all covariates tested
☐	☒	A description of any assumptions or corrections, such as tests of normality and adjustment for multiple comparisons
☐	☒	A full description of the statistical parameters including central tendency (e.g. means) or other basic estimates (e.g. regression coefficient) AND variation (e.g. standard deviation) or associated estimates of uncertainty (e.g. confidence intervals)
☐	☒	For null hypothesis testing, the test statistic (e.g. F, t, r) with confidence intervals, effect sizes, degrees of freedom and P value noted<br>*Give P values as exact values whenever suitable.*
☒	☐	For Bayesian analysis, information on the choice of priors and Markov chain Monte Carlo settings
☒	☐	For hierarchical and complex designs, identification of the appropriate level for tests and full reporting of outcomes
☒	☐	Estimates of effect sizes (e.g. Cohen's d, Pearson's r), indicating how they were calculated

Our web collection on statistics for biologists contains articles on many of the points above.

Software and code

Policy information about availability of computer code

Data collection	FACSDiva v.8.0.2 (BD Life Sciences) - flow cytometry<br>ProSort v.1.6 (BioRad) - cell sorting<br>LAS AF v.2.7.4 (Leica) - confocal microscopy<br>Typhoon FLA 9500 software v.1.1.0.187 (GE) - phosphorimaging
Data analysis	Analysis pipelines for haploid screens are available at https://github.com/BrummelkampResearch<br>ImageJ v.1.0 (NIH) - image quantification<br>FlowJo v.10.6.2 (BD Life Sciences) - flow cytometry analysis<br>PyMol v.2.5.4 (Schrodinger) - handling/representation of protein structures and models<br>Prism v.9.5.1 (GraphPad) - statistical analysis and plot construction<br>Photoshop v.24.4.1 (Adobe) - handling of microscopy images<br>RStudio v.2023.03.0+386 (Posit Software) - plot construction

For manuscripts utilizing custom algorithms or software that are central to the research but not yet described in published literature, software must be made available to editors and reviewers. We strongly encourage code deposition in a community repository (e.g. GitHub). See the Nature Portfolio guidelines for submitting code & software for further information.

March 2021

Data

Policy information about availability of data

All manuscripts must include a data availability statement. This statement should provide the following information, where applicable:

- Accession codes, unique identifiers, or web links for publicly available datasets
- A description of any restrictions on data availability
- For clinical datasets or third party data, please ensure that the statement adheres to our policy

Sequencing data and screening data will be available, respectively, at the NCBI Sequence Read Archive (www.ncbi.nlm.nih.gov.sra; under the accession numbers SAMN35570720, SAMN35570721, and SAMN35570722) and an interactive screening database (https://phenosaurus.nki.nl/) upon publication.

Human research participants

Policy information about studies involving human research participants and Sex and Gender in Research.

Reporting on sex and gender	n/a
Population characteristics	n/a
Recruitment	n/a
Ethics oversight	n/a

Note that full information on the approval of the study protocol must also be provided in the manuscript.

Field-specific reporting

Please select the one below that is the best fit for your research. If you are not sure, read the appropriate sections before making your selection.

☒ Life sciences ☐ Behavioural & social sciences ☐ Ecological, evolutionary & environmental sciences

For a reference copy of the document with all sections, see nature.com/documents/nr-reporting-summary-flat.pdf

Life sciences study design

All studies must disclose on these points even when the disclosure is negative.

Sample size	Sample sizes were determined based on previous experience and norms in the field; no sample size calculations were performed. In instances requiring statistics, the sample size is described in the figure legends, and generally is three or more replicates.
Data exclusions	No data was excluded from analysis, except for sequencing reads that, when aligned to the human genome using Bowtie, carried more than one mismatch.
Replication	The number of replicates is indicated in the figure legends. All attempts at replication were successful.
Randomization	No randomization was performed, as phenotypes were assayed based on genotype and not treatment.
Blinding	In experiments where subjectivity could be introduced (i.e. the weighing of mice, quantification of microscopy images), the experimenter was blinded to the condition under which the experiment was completed.

Reporting for specific materials, systems and methods

We require information from authors about some types of materials, experimental systems and methods used in many studies. Here, indicate whether each material, system or method listed is relevant to your study. If you are not sure if a list item applies to your research, read the appropriate section before selecting a response.

Materials & experimental systems

n/a	Involved in the study
☐	☒ Antibodies
☐	☒ Eukaryotic cell lines
☒	☐ Palaeontology and archaeology
☐	☒ Animals and other organisms
☒	☐ Clinical data
☒	☐ Dual use research of concern

Methods

n/a	Involved in the study
☒	☐ ChIP-seq
☐	☒ Flow cytometry
☒	☐ MRI-based neuroimaging

Antibodies

Antibodies used

anti-ACTB (Abcam, ab6276) WB 1:10000
anti-alpha-tubulin (Santa Cruz Biotechnology, sc-32293) WB 1:1000
anti-AMPK (Cell Signaling Technology, 2532) WB 1:1000
anti-AMPK pT172 (Cell Signaling Technology, 2535) WB 1:1000
anti-CANX (Abcam, ab22595) IF 1:100, WB 1:1000
anti-CLTC (Thermo Fisher, PA5-17347) WB 1:1000
anti-EIF4G (Cell Signaling Technology, 2498) WB 1:1000
anti-FASN (Santa Cruz Biotechnology, sc-55580) WB 1:1000
anti-HA (Biolegend, 901503) IF 1:200, WB 1:1000
anti-HSPA5 (Cell Signaling Technology, 3177) WB 1:1000
anti-LAMP1 (Santa Cruz Biotechnology, sc-19992) WB 1:1000
anti-LC3B (Cell Signaling Technology, 2775) WB 1:1000
anti-LDHA (Cell Signaling Technology, 3582) WB 1:5000
anti-PDI (Abcam, ab2792) IF 1:500, WB 1:20000
anti-S6 (Cell Signaling Technology, 2317) WB 1:1000
anti-S6 pS235/pS236 (Cell Signaling Technology, 4856) WB 1:1000
anti-TMX1 (Atlas Antibodies, HPA003085) IF 1:100, WB 1:1000
anti-TMX1 (Origene, TA507042) WB 1:1000
anti-TOMM20 (Abcam, ab186735) WB 1:10000
anti-V5 (ThermoFisher, 14-6796-82) IF 1:500, WB 1:1000

Validation

anti-ACTB (Abcam, ab6276) detects ACTB by WB in human cells, as shown by the manufacturer using HAP1 ACTB KO cells (https://www.abcam.com/products/primary-antibodies/beta-actin-antibody-ac-15-ab6276.html)

anti-alpha-tubulin (Santa Cruz Biotechnology, sc-32293) was validated by WB using purified human protein in PMID 29146869

anti-AMPK (Cell Signaling Technology, 2532) was validated by WB in mouse cells in PMID 33596428 using AMPKa KO MEFs

anti-AMPK pT172 (Cell Signaling Technology, 2535) was validated by WB in mouse cells in PMID 33596428 using AMPKa KO MEFs

anti-CANX (Abcam, ab22595) was validated by IF as an ER marker in human cells and by WB as an ER stress-responsive protein in human cells (this paper)

anti-CLTC (Thermo Fisher, PA5-17347) was validated as a cytosol/membrane marker in human cells (this paper)

anti-EIF4G (Cell Signaling Technology, 2498) detects a band of the correct size in human cells, as shown by the manufacturer (https://www.cellsignal.com/products/primary-antibodies/eif4g-antibody/2498)

anti-FASN (Santa Cruz Biotechnology, sc-55580) detects FASN by WB in human cells, as shown in PMID 32111832 using HAP1 FASN KO cells

anti-HA (Biolegend, 901503) was validated for WB and IF in human cells by the manufacturer (https://www.biolegend.com/en-us/products/purified-anti-ha-11-epitope-tag-antibody-11374)

anti-HSPA5 (Cell Signaling Technology, 3177) was validated by WB as an ER stress-responsive protein in human cells (this paper)

anti-LAMP1 (Santa Cruz Biotechnology, sc-19992) detects LAMP1 by WB in human cells, as shown in PMID 24970085 using HAP1 LAMP1 KO cells

anti-LC3B (Cell Signaling Technology, 2775) detects LC3B by WB in human cells, as shown in PMID 31208283

anti-LDHA (Cell Signaling Technology, 3582) detects a band of the correct size in human cells, as shown by the manufacturer (https://www.cellsignal.com/products/primary-antibodies/ldha-c4b5-rabbit-mab/3582)

anti-PDI (Abcam, ab2792) detects PDI (P4HB) by IF in human cells (https://www.abcam.com/products/primary-antibodies/p4hb-antibody-rl90-ab2792.html)

anti-S6 (Cell Signaling Technology, 2317) detects a band of the correct size in human cells, as shown by the manufacturer (https://www.cellsignal.com/products/primary-antibodies/s6-ribosomal-protein-54d2-mouse-mab/2317)

anti-S6 pS235/pS236 (Cell Signaling Technology, 4856) was validated by WB by the manufacturer in stimulated human cells (https://www.cellsignal.com/products/primary-antibodies/phospho-s6-ribosomal-protein-ser235-236-2f9-rabbit-mab/4856)

anti-TMX1 (Atlas Antibodies, HPA003085) was validated by WB and IF in human cells using HAP1 TMX1 KO cells (this paper)

anti-TMX1 (Origene, TA507042) was validated by WB in human cells using HAP1 TMX1 KO cells (this paper)

anti-TOMM20 (Abcam, ab186735) detects a band of the correct size in human cells, as shown by the manufacturer (https://www.abcam.com/products/primary-antibodies/tomm20-antibody-epr15581-54-mitochondrial-marker-ab186735.html)

anti-V5 (ThermoFisher, 14-6796-82) was validated for WB and IF in human cells by the manufacturer (https://www.thermofisher.com/antibody/product/V5-Tag-Antibody-clone-TCM5-Monoclonal/14-6796-82)

Eukaryotic cell lines

Policy information about cell lines and Sex and Gender in Research

Cell line source(s)	HAP1 cells were subcloned and isolated in house; they are available from Horizon Discovery. HEK293T, A549, HeLa, HT-29, RPE-1 and U2OS and U251 cells were purchased from ATCC.
Authentication	The ploidy of HAP1 cells was routinely checked by DAPI content. No other cell lines were authenticated
Mycoplasma contamination	Cell lines were routinely tested for mycoplasma and the lines/clones used in this study were consistently negative.
Commonly misidentified lines (See ICLAC register)	HEK293T

Animals and other research organisms

Policy information about studies involving animals; ARRIVE guidelines recommended for reporting animal research, and Sex and Gender in Research

Laboratory animals	This study used C57/BL6J and C57/BL6N mice, aged 2 to 28 weeks. Mice were maintained in a certified animal facility at 21°C and 55% humidity, in 12h light/dark cycles.
Wild animals	This study did not use wild animals.
Reporting on sex	Body weight measurements were carried out in both male and female mice, as this measurement is non-intrusive. Other measurements (TAG content of serum, lipidomics, immunoblots and morphological analysis) were performed in male mice as female mice were reserved for colony maintenance.
Field-collected samples	This study did not involve samples collected from the field.
Ethics oversight	National Ethics Committee for Animal Experiments of the Netherlands

Note that full information on the approval of the study protocol must also be provided in the manuscript.

Flow Cytometry

Plots

Confirm that:

☒ The axis labels state the marker and fluorochrome used (e.g. CD4-FITC).

☒ The axis scales are clearly visible. Include numbers along axes only for bottom left plot of group (a 'group' is an analysis of identical markers).

☒ All plots are contour plots with outliers or pseudocolor plots.

☒ A numerical value for number of cells or percentage (with statistics) is provided.

Methodology

Sample preparation

Haploid screens:

2-3 x 10^9 gene-trapped HAP1 cells of the indicated genotype were harvested by trypsinization and fixed in Fix Buffer I (BD Biosciences) for 10 minutes at 37°C. For the oleic acid-loaded screen, cells were first cultured for 24h in complete medium supplemented with 200 µM oleic acid, and then chased in medium lacking oleic acid for another 24h prior to harvesting. Cells were treated with 1 mg/ml RNase A (Qiagen) diluted in FACS buffer (10% FBS in PBS) at 37°C for 30 minutes prior to staining with 1 µg/ml BODIPY 493/503 and 10 µg/ml propidium iodide (Sigma-Aldrich), diluted in FACS buffer, for one hour at room temperature. Cells were washed twice in FACS buffer before being passed through a 40 µm cell strainer.

	BODIPY 493/503 measurements in fixed HAP1 cells:<br><br>HAP1 cells, grown in 10cm plates, were collected by trypsinization and were fixed in Fix Buffer I (BD Biosciences) for 10 minutes at 37°C. Cells were pelleted, washed with FACS buffer (10% FBS in PBS), resuspended in FACS buffer and counted. 10 million cells were stained with 1 µg/ml BODIPY 493/503 and 5 µg/ml DAPI (Invitrogen), diluted in FACS buffer, for one hour at room temperature. Cells were washed once in FACS buffer, then passed through a 35 µm nylon mesh cell strainer into a FACS tube.<br><br>Mitochondrial measurements in live RPE1 cells:<br><br>RPE1 cells were treated as described and pulsed for 30 minutes with either 600 nM TMRM or 250 nM MitoTracker Red CM-H2XROS in medium depleted of lipoproteins. In TMRM experiments, cells were then incubated in 150 mM for an additional 30 minutes, using 20 µM CCCP as a positive control. Cells were collected by trypsinization and stored on ice in FACS buffer.
Instrument	Cell sorting for screens was performed using an S3 Sorter (Bio-Rad) and analytical flow cytometry was performed on an LSR Fortessa (BD Biosciences).
Software	Haploid screens: sorting was performed using ProSort (Bio-Rad)<br>Analytical flow cytometry: flow cytometry was performed using FACSDiva (BD) and data was analyzed using FlowJo (BD)
Cell population abundance	For haploid screens, 10^7 cells were collected for both the lowest and highest 5% of BODIPY signal from haploid cells in G1. Prior to genomic DNA isolation, several thousand cells from each sorted population were re-analyzed to ensure purity of the sorted cells.
Gating strategy	FSC-A and SSC-A were gated to exclude cell debris. For haploid screens, PI (area) channel was gated for single haploid cells in G1 (DNA content = 1n). From this population, BODIPY 493/503 (area) was assessed using a 488 nm laser.<br><br>Intact RPE1 cells were identified using FSC-A/SSC-A as above, and then singlets were identified by SSC-A/SSC-W. From this population, mean fluorescence was assessed.

☒ Tick this box to confirm that a figure exemplifying the gating strategy is provided in the Supplementary Information.
