# Supplementary material for: Identification of an alternative triglyceride biosynthesis pathway
Source: Nature. 2023 Aug 30;621(7977):171–8. doi: 10.1038/s41586-023-06497-4 (PMC10482677; doi:10.1038/s41586-023-06497-4)
Supplement: Supplementary file 3 — Tables and their legends. [file 41586_2023_6497_MOESM3_ESM.docx]

**SUPPLEMENTARY INFORMATION – Supplementary Tables**

Supplementary Table 1. Synthetic guide RNA sequences and targets

| target gene (sgRNA) | target exon | sgRNA target sequence (5’-3’) |
| --- | --- | --- |
| control | - | GGTATGTCGGGAACCTCTCC |
| *DGAT1* | 2 | AGTGGCTTCAGCAACTACCG |
| *DGAT2* (#1) | 3 | TGTGCTCTACTTCACTTGGC |
| *DGAT2* (#2) | 4 | GGGTCCGAAACTGGGCTGTG |
| *DIESL* (#1) | 4 | CAAAGTGATCAGCTACTACT |
| *DIESL* (#2) | 4 | GCTAAAATATTTATACACAA |
| *TMX1* | 1 | CTCCGCCGCCCGTGCGTCCA |

Table summarizing sgRNAs (expressed as the 20-nucleotide target sequence upstream of the PAM sequence) used to target the indicated genetic locus.

**Supplementary Table 2. Primers used to amplify genetic loci in human cells**

| gene | exon(s) | direction | primer sequence (5’-3’) |
| --- | --- | --- | --- |
| *DGAT1* | 2 | sense | CTTTGCCCACTGTAGGTCTTGAG |
| *DGAT1* | 2 | antisense | GTAGCACAGGAAGCTCTTCTCACATC |
| *DGAT2* | 3 and 4 | sense | GTAGCTATTTCTCAGGGTTTGTGGG |
| *DGAT2* | 3 and 4 | antisense | CTTCCCTGTGGGTGGGTTAAG |
| *DIESL* | 4 | sense | CCTAATGGCTGTAGGACTGATTTGTTC |
| *DIESL* | 4 | antisense | GTTATACACACCCTAAAAGGCAAAAAGC |
| *TMX1* | 1 | sense | GTAGAAGCAGCCGCTTTTCTGTCATG |
| *TMX1* | 1 | antisense | CTTAACATTCACGGGTAAGAGACGAG |

Table summarizing primers used to amplify genetic loci in order to study genetic modifications in mutated cell lines.

**Supplementary Table 3. Mutant cell lines**

| gene | exon | mutation (5’-3’) | indel |
| --- | --- | --- | --- |
| **HAP1 *DGAT* DKO clone 1** | | | |
| *DGAT1* | 2 | -- BLAST resistance integration -- | large ins. |
| *DGAT2* | 3 | wt CGCTGTGCTCTACTTCACTTGGCTGGTGTTTG  mut CGCTGTGCTCTACT::::::::::GGTGTTTG | ∆10 bp |
| **HAP1 *DGAT* DKO clone 2** | | | |
| *DGAT1* | 2 | -- BLAST resistance integration -- | large ins. |
| *DGAT2* | 4 | -- BLAST resistance integration -- | large ins. |
| **HAP1 *∆TMX1* clone 1** | | | |
| *TMX1* | 1 | -- BLAST resistance integration -- | large ins. |
| **HAP1 *∆TMX1* clone 2** | | | |
| *TMX1* | 1 | -- BLAST resistance integration -- | large ins. |
| **HAP1 *DGAT TMX1* 3KO clone 1** | | | |
| *DGAT1* | 2 | -- BLAST resistance integration -- | large ins. |
| *DGAT2* | 4 | -- BLAST resistance integration -- | large ins. |
| *TMX1* | 1 | wt TGGGGTGCTCCCTGGA CGCACGGGCGGCGGAG  mut TGGGGTGCTCCCTGGAACGCACGGGCGGCGGAG | +1 bp |
| **HAP1 *DGAT TMX1* 3KO clone 2** | | | |
| *DGAT1* | 2 | -- BLAST resistance integration -- | large ins. |
| *DGAT2* | 4 | -- BLAST resistance integration -- | large ins. |
| *TMX1* | 1 | wt TGGGGTGCTCCCTGGA CGCACGGGCGGCGGAG  mut TGGGGTGCTCCCTGGAACGCACGGGCGGCGGAG | +1 bp |
| **HAP1 *∆DIESL* clone 1** | | | |
| *DIESL* | 4 | -- BLAST resistance integration -- | large ins. |
| **HAP1 *∆DIESL* clone 2** | | | |
| *DIESL* | 4 | wt ATATTTATAC ACAAAGGCAGA  mut ATATTTATACTGGCT...AGAAT:CAAAGGCAGA | +160 bp |
| **HAP1 *DIESL TMX1* DKO clone 1** | | | |
| *DIESL* | 4 | wt CTAAAATATTTATACA CAAAGGCAGAACTTGC  mut CTAAAATATTTATACAACAAAGGCAGAACTTGC | +1 bp |
| *TMX1* | 1 | -- BLAST resistance integration -- | large ins. |
| **HAP1 *DIESL TMX1* DKO clone 2** | | | |
| *DIESL* | 4 | wt CTAAAATATTTATACA CAAAGGCAGAACTTGC  mut CTAAAATATTTATACAACAAAGGCAGAACTTGC | +1 bp |
| *TMX1* | 1 | -- BLAST resistance integration -- | large ins. |
| **HAP1 *DGAT DIESL* 3KO clone 1** | | | |
| *DGAT1* | 2 | -- BLAST resistance integration -- | large ins. |
| *DGAT2* | 4 | -- BLAST resistance integration -- | large ins. |
| *DIESL* | 4 | wt CTAAAATATTTATACA CAAAGGCAGAACTTGC  mut CTAAAATATTTATACAACAAAGGCAGAACTTGC | +1 bp |
| **HAP1 *DGAT DIESL TMX1* 4KO clone 1** | | | |
| *DGAT1* | 2 | -- BLAST resistance integration -- | large ins. |
| *DGAT2* | 4 | -- BLAST resistance integration -- | large ins. |
| *DIESL* | 4 | wt CTAAAATATTTATACA CAAAGGCAGAACTTGC  mut CTAAAATATTTATACAACAAAGGCAGAACTTGC | +1 bp |
| *TMX1* | 1 | wt TGGGGTGCTCCCTGGA CGCACGGGCGGCGGAG  mut TGGGGTGCTCCCTGGAACGCACGGGCGGCGGAG | +1 bp |
| **293T *∆TMX1* clone 1** | | | |
| *TMX1* | 1 | wt TGGGGTGCTCCCTGGA CGCACGGGCGGCGGAG  mut TGGGGTGCTCCCTGGAACGCACGGGCGGCGGAG  mut TGGGGTGCTCCCTGGAACGCACGGGCGGCGGAG  mut -- PURO resistance integration -- | +1 bp  +1 bp  large ins. |
| **293T *∆TMX1* clone 2** | | | |
| *TMX1* | 1 | wt TGGGGTGCTCCCTGGA CGCACGGGCGGCGGAG  mut TGGGGTGCTCCCTGGAACGCACGGGCGGCGGAG  mut TGGGGTGCTCCCTGGAACGCACGGGCGGCGGAG  mut -- PURO resistance integration -- | +1 bp  +1 bp  large ins. |

Catalogue of mutated loci from cell lines used in this study. Insertions and deletions (indels) were determined from Sanger sequencing. Although this is not indicated in the table, in instances where a blasticidin (BLAST) or puromycin (PURO) resistance cassette was inserted at the indicated locus, integrations were also confirmed via sequencing as well. Genetic modifications are presented in a red font, with deletions represented as “:”. The *TMX1* locus in 293T cells are inferred to be triploid and diploid, respectively, as several other clones (not used in this study) were ascertained to be compound heterozygotes by TIDE analysis.

**Supplementary Table 4. Genetic screens**

| screen | sorted population | number of unique mutations |
| --- | --- | --- |
| unloaded HAP1 *DGAT* DKO  (Fig. 1b) | high | 3.0E6 |
|  | low | 3.5E6 |
| oleic acid-loaded HAP1 WT  (Fig. 2c) | high | 7.7E6 |
|  | low | 6.4E6 |
| unloaded HAP1 *∆TMX1*  (Fig. 2c) | high | 6.1E6 |
|  | low | 5.8E6 |

Table summarizing the number of unique mutations (gene-trap integrations) in the indicated FACS-sorted population for the haploid genetic screens contained within this study.
